# SELF-REPORTED DISCRIMINATION AND BRAIN STRUCTURAL ABNORMALITIES: THE MULTI-ETHNIC STUDY OF ATHEROSCLEROSIS

**DOI:** 10.1093/geroni/igae098.1309

**Published:** 2024-12-31

**Authors:** Sarah Forrester, Jordan Tanley, Michael Bancks, José Luchsinger, Stephen Rapp, Marcia P Jiminez, Robert Bryan, Timothy Hughes

**Affiliations:** University of Massachussetts Chan Medical School, Worcester, Massachusetts, United States; Wake Forest University School of medicine, Winston-Salem, North Carolina, United States; Wake Forest University School of Medicine, Winston-Salem, North Carolina, United States; Columbia University, New York, New York, United States; Wake Forest University School of Medicine, Winston-Salem, North Carolina, United States; Boston University, Boston, Massachusetts, United States; University of Pennsylvania, Philadelphia, Pennsylvania, United States; Wake Forest University School of Medicine, Winston-Salem, North Carolina, United States

## Abstract

Black and Hispanic adults have greater risk for developing Alzheimer’s disease (AD) and related dementias (ADRD) compared to White adults. These disparities might be at least partially attributable to adverse social determinants of health (SDoH) such as experienced interpersonal discrimination. This study examines racial/ethnic differences in the association between self-reported discrimination and brain imaging findings associated with aging and AD/ADRD. We used data from the MESA study, which initially enrolled 6814 participants in 2000-02. when they self-reported their race/ethnicity (38% White, 28% Black, 22% Hispanic, 12% Chinese Americans) and instances of lifetime discrimination. The primary exposure was experiencing racial discrimination. During Exam 6 (2016-18) and ancillary study MIND A (2019-21), brain imaging with 3-Tesla MRI (n=1430) and amyloid PET (n=315) was completed. Multivariable linear (brain MRI measures) and logistic (amyloid PET positivity [centiloid >12.2]) regression models were adjusted for covariates including age, race/ethnicity, gender, education, site, smoking status, blood pressure, diabetes, APOE-ε4, income, health insurance, (all models) and intracranial volume (for brain volumes). Race/ethnicity stratified analyses were completed to assess moderation. Racial discrimination was positively associated with greater white matter hyperintensity volume (WMHV), but not gray matter volume (GMV) or fractional anisotropy (p = 0.008). Racial discrimination was also significantly associated with higher odds of amyloid positivity [OR (95%CI) = 2.24(1.07, 4.70)]. Stratified analysis revealed stronger associations between racial discrimination and GMV and WMHV in Black participants (p = 0.008 and 0.003, respectively). Instances of racial discrimination over a lifetime may be detrimental to brain health, especially for Black adults.

